# Characterisation of the inflammatory cytokine and growth factor profile in a rabbit model of proliferative vitreoretinopathy

**DOI:** 10.1038/s41598-019-51633-8

**Published:** 2019-10-28

**Authors:** Chee Wai Wong, Ning Cheung, Candice Ho, Veluchamy Barathi, Gert Storm, Tina T. Wong

**Affiliations:** 1 Singapore National Eye Centre (SNEC), 11 Third Hospital Avenue, Singapore, 168751 Singapore; 2 Singapore Eye Research Institute, 11 Third Hospital Avenue, Singapore, 168751 Singapore; 3 Department Pharmaceutics, Utrecht Institute for Pharmaceutical Sciences (UIPS), Utrecht University, PO Box 80082, 3508 TB Utrecht, The Netherlands; 4 School of Materials Science and Engineering (MSE), Nanyang Technological University, 11 Faculty Avenue, Singapore, 639977 Singapore; 5 Ophthalmology and Visual Sciences Academic Clinical Program, Duke NUS Medical School, 8 College Rd, Singapore, 169857 Singapore; 6 Department Biomaterials Science & Technology (BST), Section Targeted Therapeutics, University of Twente, PO Box 217, 7500 AE Enschede, The Netherlands

**Keywords:** Prognostic markers, Retinal diseases

## Abstract

To clarify the mechanisms and their temporal relationship in the development of proliferative vitreoretinopathy (PVR), we measured vitreous levels of pro-inflammatory cytokines and growth factors in a rabbit model of PVR. PVR was surgically induced in 11 rabbit eyes by vitrectomy, retinotomy, cryotherapy and injection of platelet-rich plasma at baseline. Severity of PVR was assessed on dilated fundal examination with indirect binocular ophthalmoscopy and graded based on the revised experimental PVR classification. Severe PVR was defined as stage 5 or worse. Vitreous concentrations of interleukin 6 (IL-6), interleukin 8 (IL-8), interleukin 1 beta (IL-1 β), tumor necrosis factor beta (TNF-β), granulocyte macrophage colony stimulating factor (GM-CSF), interferon gamma (IFN-γ), C reactive protein; (CRP), placental growth factor (PlGF), platelet derived growth factor BB (PDGF-BB), vascular endothelial growth factor (VEGF) and angiopoietin 2 (Ang-2) at weeks 2, 3 and 4 were compared to baseline and correlations between the cytokines with PVR severity were assessed. Four weeks after PVR induction, 5 eyes (45.5%) had developed severe PVR. IL-8 was raised at 2 weeks post PVR induction (1.46 ± 0.48 pg/ml vs 0.53 ± 0.25 pg/ml, p = 0.04) and remained significantly elevated at week 4 (2.6 ± 3.1 pg/ml, p = 0.03). CRP was significantly raised at week 4 (34.8 ± 12.0 pg/ml vs 13.0 ± 13.1 pg/ml, p < 0.001). Among the growth factors, PDGF-BB was the earliest to show significantly elevated levels, at 3 weeks (50.4 ± 19.0 pg/ml vs 6.2 ± 10.1 pg/ml) and remained elevated at week 4 (p = 0.002), while PlGF (11.2 ± 7.7 pg/ml vs 5.3 ± 3.8 pg/ml, p = 0.002) and Ang2 (13617.0 ± 8170.2 pg/ml vs 38593.8 ± 8313.4, p = 0.02) were significantly raised at week 4. IFN-γ (p = 0.03), PDGF-BB (p = 0.02) and VEGF (p = 0.02) were significantly associated with PVR severity. We demonstrated that inflammatory cytokines IL-6, -8, elevation post PVR induction is followed by elevated levels of fibroproliferative growth factors, Ang2, PlGF, VEGF and PDGF-BB in the development of PVR. These findings will guide future studies targeting appropriate therapeutic strategies for the treatment of PVR.

## Introduction

Proliferative vitreoretinopathy (PVR) is a blinding condition that can occur secondary to penetrating ocular trauma, retinal detachment, or following surgery for retinal detachment repair. In these conditions, a breach in the integrity of the retina introduces macrophages, retinal pigment epithelial cells, glial cells, and fibroblasts into the vitreous, where they proliferate and incite inflammation. This process has been likened to keloidal scar formation, which in the eye can result in massive retinal detachment, scarring and obliteration of vision^1^. PVR is the most common reason for failure of retinal detachment (RD) surgery: anatomical success rates in RD complicated by PVR is only 69–75% compared to 98% in RD without PVR, and visual outcomes of this surgery are worse when complicated by PVR^2,3^. Although surgery is the mainstay of treatment for RD complicated by PVR, multiple surgeries are frequently required to eventually achieve final retinal attachment often with unsatisfactory visual outcomes^3^. In addition, following RD surgery, patients with PVR require twice as many care resources compared to patients without PVR. These resources include not only the economic burden of multiple surgeries but also a longer time spent recovering from surgery and thus away from employment, longer follow up duration and increased patient burden for the hospital, as well as the emotional burden of poor visual outcomes for both patients and their caretakers^3^.

For the past 40 years, many pharmacological agents have shown promising results in animal models of PVR but none have successfully achieved clinical application due to limited efficacy in humans. This failure to translate preclinical success can perhaps be attributed to 2 main reasons. First, there is a lack of clarity in the pathogenesis of PVR. This in turn led to the development of inappropriate animal models that do not reflect the disease process and therefore led to a lack of clinical efficacy for therapeutic agents tested using these models. Second, PVR is a multi-stage disease involving many pathogenic pathways. These pathways can be broadly grouped into inflammation, proliferation and epithelial mesenchymal transition processes. A single agent cannot be expected to be efficacious in all multiple cellular processes that together make up the clinical complication. Instead, a multi-agent therapeutic strategy directed at the correct targets at the correct time should be the approach of choice for treating such a complex disease.

To help clarify the mechanisms and their temporal relationship in the development of PVR, we conducted a study of changes in cytokine levels following surgically induced PVR in the rabbit. This rabbit model is a surgical model based on human pathogenesis, i.e. PVR following retinal detachment and thus reflects clinically relevant disease. The aim of this study was to compare the levels of pro-inflammatory cytokines and growth factors involved in RPE cell proliferation and epithelial mesenchymal transition at various time points in the evolution of PVR, and to correlate these levels to the severity of PVR.

## Materials and Methods

### Animals

The SingHealth Institute Animal Care and Use Committee (IACUC Singhealth Approval Number 2016/SHS/1256) approved this study. All procedures conducted in this study complied with the ARVO Statement for the Use of Animals in Ophthalmic and Vision Research. 11 New Zealand White adult rabbits, with weights of 2–2.5 kg were used in this study. Only rabbits with no ocular disease as confirmed on slit lamp examation were included in the study.

### Induction of PVR

After sterilizing the eye with 5% iodine solution, 23-gauge trans pars plana vitrectomy (Stellaris PC, Bausch and Lomb, Irvine, CA) was performed^4,5^. Four retinotomies, of 500um (one third disc diameter) in size each were performed with a 41 gauge needle and bleb retinal detachments of 3–4 disc diameters were induced by injection of balanced salt solution at 4 separate sites in the inferior retina. Rabbits received an intravitreal injection, using a 25-gauge needle into the central vitreous, 4 mm behind the limbus of 0.1 ml platelet rich plasma (PRP). PRP was prepared from rabbit homologous blood according to the method of Constable *et al*.^6^. Pooled arterial blood was collected from the rabbit’s ear artery into plastic tubes containing an anticoagulant solution (1 part 3.8% sodium citrate to 9 parts whole blood). This fresh citrated blood was centrifuged at 1,200 rotations per min for 10 min, and the upper third of the supernatant PRP was aspirated. Tobramycin eyedrops were instilled into the eye 4 times a day for 5 days after induction of PVR.

### Investigations and examination

The retinal status was examined with an indirect ophthalmoscope through a +20 D fundus lens on days 1, 7, 14, 21 and 28. by two double-masked ophthalmologists (CWW, DC). PVR was graded according to the revised PVR classification:

Revised PVR classification^7^

Stage 0: Normal retina (A)

Stage 1: Surface wrinkling (B)

Stage 2: Mild pucker (C)

Stage 3: Severe pucker (D)

Stage 4: Elevated pucker (E)

Stage 5: Partial retinal detachment (F)

Stage 6: Low detachment (G)

Stage 7: Total detachment (H)

Severe PVR was defined as stage 5 or worse PVR. Fundus photographs were taken with a 45-degree digital retinal camera after pupillary dilation with tropicamide1%, using Canon CR-DGi with Canon EOS 10D SLR backing (Canon Inc, Tokyo, Japan).

### Collection of vitreous samples and analysis

Vitreous humor samples were obtained at baseline during the start of the vitrectomy procedure. At day 14 and 21, vitreous humor samples of 0.2 ml each were obtained with a 23 G needle on a 5 ml syringe via the pars plana. On day 28, eyes were enucleated and the vitreous obtained prior to paraffin fixation of the eye. Vitreous samples were stored at −80 degrees Celsius prior to analysis. The vitreous concentrations of interleukin 6 (IL-6), interleukin 8 (IL-8), interleukin 1 beta (IL-1β), tumor necrosis factor beta (TNF-β), granulocyte macrophage colony stimulating factor (GM-CSF), interferon gamma (IFN-γ), C reactive protein; (CRP), placental growth factor (PlGF), platelet derived growth factor BB (PDGF-BB), vascular endothelial growth factor (VEGF) and angiopoietin 2 (Ang-2) were determined using the Human multiplex ELISA kit from AYOXXA

### Enucleation, euthanasia and pathology procedures

Euthanization was carried out on all rabbits at the end of the 28 day study period with intraperitoneal pentobarbitone (60–150 mg/kg). The study eyes were then enucleated.

### Histopathology and immunohistochemistry

The procedures performed for histology and immunohistochemistry have been previously described by our group^8^. Eyes were enucleated and fixed in a mixture of 10% neutral buffered formalin solution (Leica Surgipath, Leica Biosystems Richmond, Inc.) for 24 hours. The whole eye were then dissected to anterior and posterior segment prior to dehydration in increasing concentration of ethanol, clearance in xylene, and embedding in paraffin (Leica-Surgipath, Leica Biosystems Richmond, Inc.) Four-micron sections were cut with a rotary microtome (RM2255, Leica Biosystems Nussloch GmbH, Germany) and collected on POLYSINE^TM^ microscope glass slides (Gerhard Menzel, Thermo Fisher Scientific, Newington, CT). The sections were dried in an oven of 37 °C for at least 24 hour. To prepare the sections for histopathological and immunohistochemical examination, the sections were heated on a 60 °C heat plate, deparaffinized in xylene and rehydrated in decreasing concentration of ethanol. A standard procedure for Hematoxylin and Eosin (H&E) was performed. A light microscope (Axioplan 2; Carl Zeiss Meditec GmbH, Oberkochen, Germany) was used to examine the slides and images were captured (Table 1).

**Table 1:** Antibodies used for immunohistochemical staining.

Antibody	Catalog No.	Company	Concentration
Smooth muscle actin	710487	Thermo fisher Scientific	1:200
GFAP GA5	14-9892-82	Thermo fisher Scientific	1:200
Vimentin	MA511883	Thermo fisher Scientific	1:200
Alexa Fluor 488 goat anti−mouse IgG (H + L)	A11001	Invitrogen. Life Technologies (Invitrogen, Eugene, OR)	1:1000

In parallel, immunofluorescence staining was performed. Heat-induced antigen retrieval was performed by incubating sections in sodium citrate buffer (10 mM Sodium citrate, 0.05% Tween 20, pH 6.0) for 20 minutes at 95–100 °C. The sections were then cooled down in sodium citrate buffer for 20 minutes in RT and washed three times for 5 minutes each with 1X PBS. Non-specific sites were blocked with blocking solution of 5% bovine serum albumin (BSA) in 0.1% Triton X-100 and 1XPBS for 1 hour at room temperature in a humidified chamber. The slides were then rinsed briefly with 1X PBS. A specific primary antibody shown in Table 1 was applied and incubated overnight at 4 °C in a humidified chamber prepared in blocking solution. After washing twice with 1XPBS and once with 1X PBS with 0.1% tween for 10 minutes each, Alexa Fluro® 488 – conjugated fluorescein-labeled secondary antibody shown in Table 1 (Invitrogen- Molecular Probes, Eugene, OR) was applied at a concentration of 1:1000 in blocking solution and incubated for 90 minutes at RT. The slides were then washed twice with 1XPBS and once with 1X PBS with 0.1% tween for 5 minutes each, the slides were mounted on the slides with Prolong Diamond Anti-fade DAPI5 Mounting Media (Invitrogen- Molecular Probes, Eugene, OR) to visualize cell nucleic. For negative controls, primary antibody was omitted.

A fluorescence microscope (Axioplan 2; Carl Zeiss Meditec GmbH, Oberkochen, Germany) was used to examine the slides and images were captured. Experiments were repeated in duplicates for the antibody.

### Statistical analysis

Statistical analysis was performed with Stata 13.0 (Stata Corporation, College Station, TX). Continuous data were presented as mean ± standard deviation (SD). Cytokine levels were compared with the paired t test while proportions were analysed with the chi square test. The *P*-value for trend across time from PVR induction were calculated, and multivariable analysis was performed to assess associations of cytokine levels with PVR severity, using ordinal logistic regression adjusted for time from PVR induction. A two-tailed p value of < 0.05 was considered statistically significant.

## Results

### PVR severity

**Figure 1:**
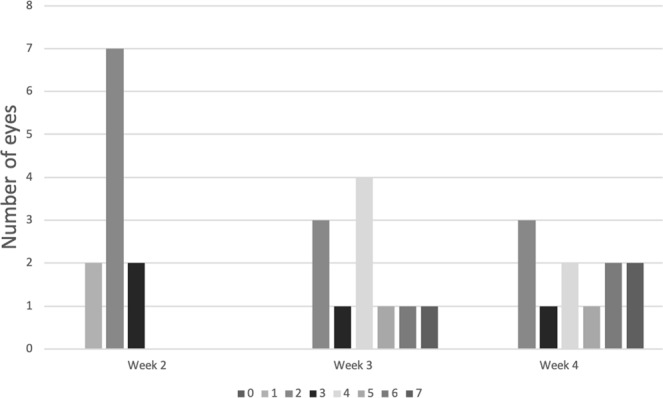
Number of eyes in each stage of PVR severity at different time intervals after PVR induction.

**Figure 2:**
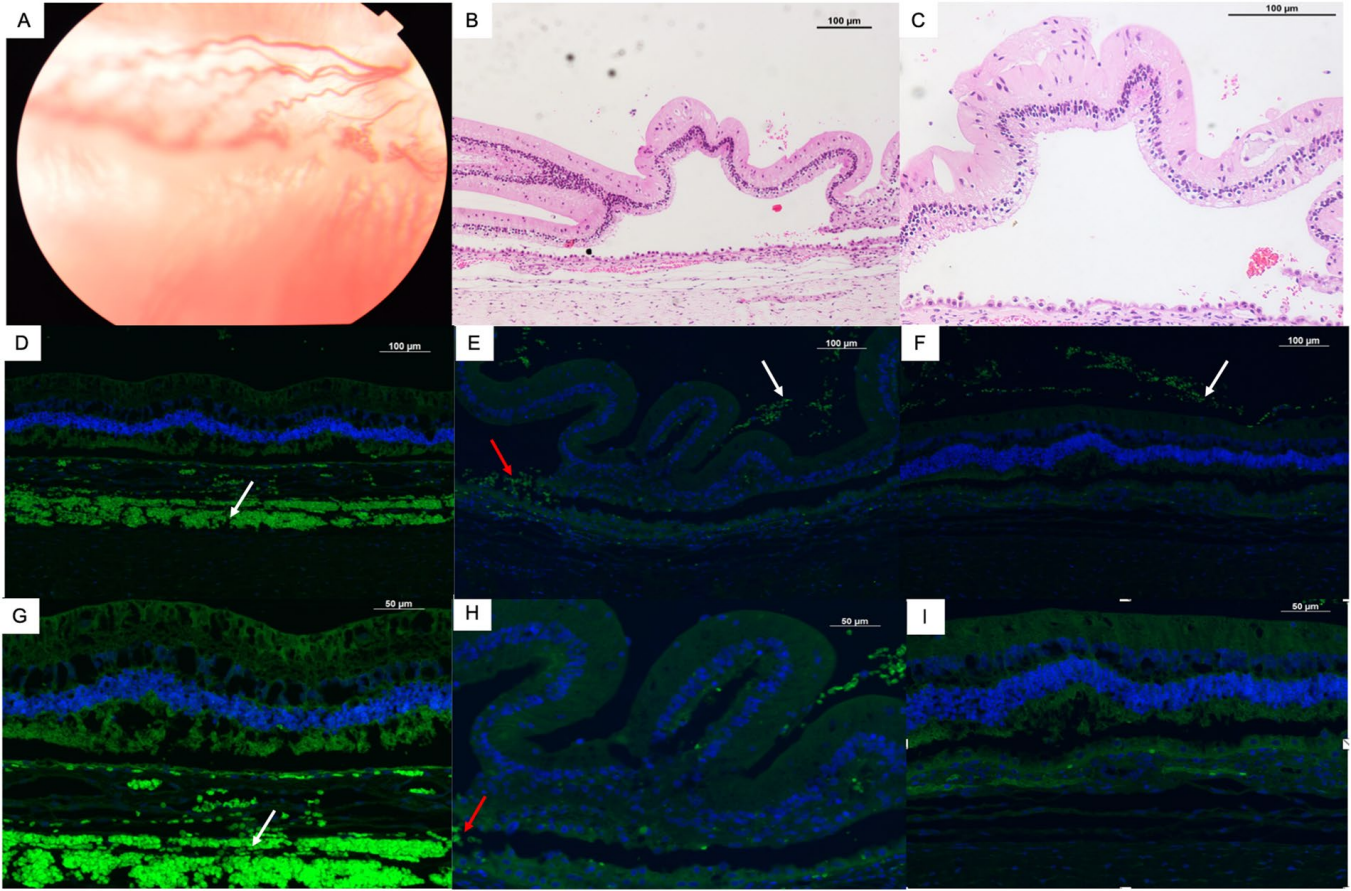
Fundus photo, H&E staining and immunohistochemical staining of a rabbit eye with severe PVR. (**A**) Fundus photo shows partial detachment of the retina with folding of the detached retina. Detachment and folding of the retina can be observed in detail on H&E staining at 10X (**B**) and 20X (**C**) magnification. Epiretinal membranes (white arrows) stained positively with alpha smooth muscle actin (**E**) and glial fibrillary acid protein (**F**), demonstrating the presence of glial and mesenchymal elements in these membranes. Vimentin staining was present within the subretinal space (**D** and **G**, white arrow) and smooth muscle actin staining was present in subretinal membranes as well (**E** and **H**, red arrows). (**G**, **H** and **I**) Show vimentin, alpha smooth muscle actin and glial fibrillary acid protein staining at 20X magnification.

Figure 1 shows the distribution of PVR severity across the study period. At 2 weeks after PVR induction, most eyes (n = 7, 63.6%) had developed at least stage 1 PVR and none had severe PVR. There is an increase in number of eyes with severe PVR (Fig. 2A) at week 3 (n = 3, 27.2%)). Four weeks after PVR induction, 5 eyes (45.5%) had developed severe PVR.

### Cytokine and growth factor levels

Table 2 shows the mean cytokine levels at baseline, weeks 2,3 and 4 post PVR induction. IL-8 was significantly raised at 2 weeks post PVR induction (1.46 ± 0.48 pg/ml vs 0.53 ± 0.25 pg/ml, p = 0.04) and remained significantly elevated at week 4 (2.6 ± 3.1 pg/ml, p = 0.03). CRP was significantly raised at week 4 (34.8 ± 12.0 pg/ml vs 13.0 ± 13.1 pg/ml, p < 0.001).

**Table 2:** Mean cytokine and growth factor concentrations in the vitreous from baseline to week 4.

Molecular factor levels, pg/ml	Baseline	Week 2	P*	Week 3	P*	Week 4	P*	P trend
IL-6	0.39 ± 0.25	0.59 ± 0.24	0.38	0.67 ± 0.23	0.32	0.72 ± 0.57	0.06	0.008
IL-8	0.53 ± 0.25	1.46 ± 0.48	0.04	11.5 ± 17.0	0.22	2.6 ± 3.1	0.03	<0.001
IL-1β	0.20 ± 0.05	0.24 ± 0.06	0.58	0.46 ± 0.13	0.11	0.37 ± 1.90	0.12	0.13
TNF-β	2.1 ± 0.79	3.23 ± 1.05	0.47	3.59 ± 0.54	0.29	0.37 ± 0.13	0.12	0.71
GM-CSF	0.29 ± 0.05	0.33 ± 0.06	1.00	0.39 ± 0.03	0.29	0.29 ± 0.07	0.89	0.75
IFN-γ	3.8 ± 1.77	5.07 ± 1.53	1.00	9.28 ± 1.20	0.29	6.08 ± 2.65	0.14	0.21
CRP	13.0 ± 13.1	30.7 ± 12.1	0.22	35.9 ± 9.8	0.07	34.8 ± 12.0	0.0008	0.001
PlGF	5.3 ± 3.8	9.0 ± 3.8	0.66	13.2 ± 5.2	0.05	11.2 ± 7.7	0.02	0.02
PDGF-BB	6.2 ± 10.1	26.2 ± 8.4	0.32	50.4 ± 19.0	0.0009	34.6 ± 27.4	0.002	0.001
VEGF	215.1 ± 145.5	311.9 ± 188.0	0.58	1875.5 ± 1356.8	0.11	1251.5 ± 1876.0	0.13	0.02
Ang2	13617.0 ± 8170.2	106464.6 ± 57182.56	0.18	24064.2 ± 10409.9	0.47	38593.8 ± 8313.4	0.02	0.007

*compared to baseline

Abbreviations:

IL-6, interleukin 6; IL-8, interleukin 8; IL-1β, interleukin 1 beta; TNF-β, tumor necrosis factor beta; GM-CSF, granulocyte macrophage colony stimulating factor; IFN-γ, interferon gamma; CRP, C reactive protein; PlGF, placental growth factor; PDGF-BB, platelet derived growth factor BB; VEGF, vascular endothelial growth factor; Ang2, angiopoietin 2.

PDGF-BB was the earliest to show significantly elevated levels, at 3 weeks (50.4 ± 19.0 pg/ml vs 6.2 ± 10.1 pg/ml) and remained elevated at week 4 (p = 0.002). PlGF (11.2 ± 7.7 pg/ml vs 5.3 ± 3.8 pg/ml, p = 0.002) and Ang2 (13617.0 ± 8170.2 pg/ml vs 38593.8 ± 8313.4, p = 0.02) were significantly raised at week 4.

IL-6, IL-8, CRP, PDGF-BB, PGF, VEGF and Ang2 all showed significant trend for elevation over the 4 weeks experimental duration.

### Association of cytokine and growth factor levels with PVR severity

Tables 3, 4 shows the association of cytokine levels with severity of PVR. Comparing cytokine levels between eyes with and without severe PVR (defined as PVR stage 5 or worse, Table 3), we found significantly higher CRP (p = 0.03), PDGF-BB (p = 0.01) and VEGF (p = 0.003) levels in eyes with severe PVR. The association of cytokine levels across all stages of PVR severity are presented in Table 4. IFN-γ (p = 0.03), CRP (p = 0.001), PDGF-BB (p < 0.001) and VEGF (p = 0.002) were significantly associated with PVR severity. After adjusting for time from PVR induction, IFN-γ (p = 0.03), PDGF-BB (p = 0.02) and VEGF (p = 0.02) remained significantly associated with PVR severity.

**Table 3:** Comparison of intravitreal cytokine and growth factor levels between eyes with and without severe proliferative vitreoretinopathy.

Molecular factor, pg/ml	With severe PVR	Without severe PVR	p
IL-6	0.51 ± 0.17	0.39 ± 0.31	0.37
IL-8	2.15 ± 1.92	2.45 ± 8.22	0.93
IL-1β	1.15 ± 1.51	0.42 ± 0.71	0.13
TNF-β	8.26 ± 10.49	4.13 ± 5.13	0.23
GM-CSF	0.32 ± 0.02	0.31 ± 0.12	0.19
IFN-γ	6.29 ± 0.79	5.15 ± 4.57	0.68
CRP	36.61 ± 7.70	21.17 ± 16.16	0.03
PlGF	45.34 ± 47.89	25.50 ± 77.34	0.55
PDGF-BB	51.63 ± 30.55	22.45 ± 24.01	0.01
VEGF	170462 ± 287247	14489 ± 53632	0.003
Ang2	71064 ± 74155	32486 ± 57994	0.16

Abbreviations:

PVR, proliferative vitreoretinopathy; IL-6, interleukin 6; IL-8, interleukin 8; IL-1β, interleukin 1 beta; TNF-β, tumor necrosis factor beta; GM-CSF, granulocyte macrophage colony stimulating factor; IFN-γ, interferon gamma; CRP, C reactive protein; PlGF, placental growth factor; PDGF-BB, platelet derived growth factor BB; VEGF, vascular endothelial growth factor; Ang2, angiopoietin 2.

Severe PVR was defined as PVR grade 5 or worse.

**Table 4:** Association of cytokines and growth factors with PVR severity.

Molecular factor	unadjusted OR	95% CI	p	adjusted OR	95% CI	p
IL-6	2.66	0.38–18.63	0.32	0.30	0.03–3.17	0.32
IL-8	1.04	0.97–1.11	0.24	1.03	0.97–1.10	0.37
IL-1β	1.33	0.65–2.71	0.44	1.05	0.43–2.57	0.92
TNF-β	1.02	0.92–1.13	0.69	0.99	0.87–1.13	0.93
GM-CSF	257.87	0.07–94000	0.19	2300000	0.64–8.41e + 12	0.06
IFN-γ	1.34	1.03–1.75	0.03	1.68	1.05–2.69	0.03
CRP	1.09	1.04–1.15	0.001	1.03	0.96–1.10	0.39
PlGF	1.00	0.99–1.01	1.00	1.01	0.99–1.02	0.40
PDGF-BB	1.06	1.03–1.09	<0.001	1.04	1.01–1.08	0.02
VEGF	1.000109	1.000039–1.000179	0.002	1.000088	1.000017–1.000159	0.02
Ang2	1.000004	0.9999948–1.000014	0.38	1.00	1.00–1.00	0.96

Abbreviations:

IL-6, interleukin 6; IL-8, interleukin 8; IL-1β, interleukin 1 beta; TNF-β, tumor necrosis factor beta; GM-CSF, granulocyte macrophage colony stimulating factor; IFN-γ, interferon gamma; CRP, C reactive protein; PlGF, placental growth factor; PDGF-BB, platelet derived growth factor BB; VEGF, vascular endothelial growth factor; Ang2, angiopoietin 2.

### Histology and immunohistochemistry

**Figure 3:**
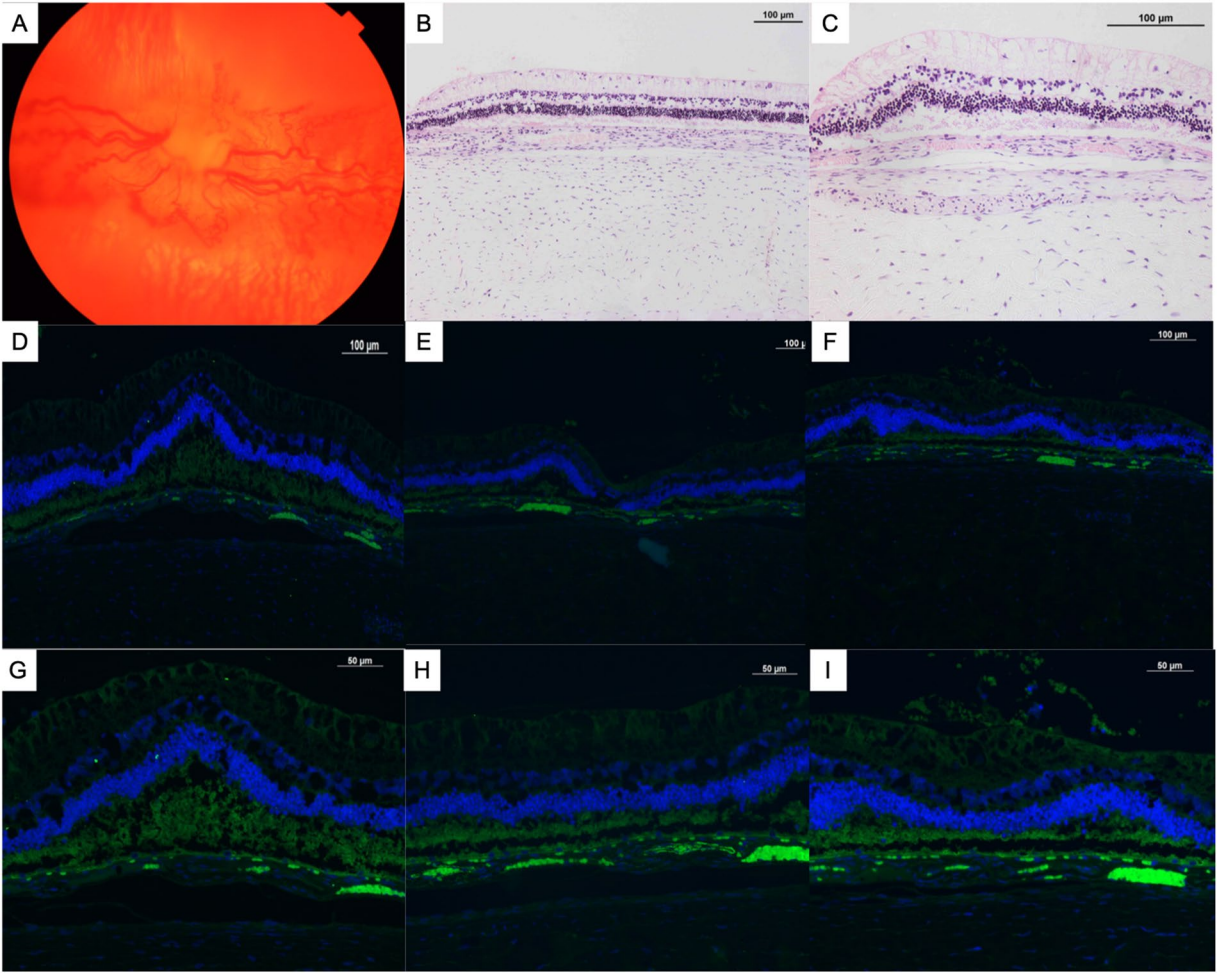
Fundus photo, H&E staining and immunohistochemical staining of a rabbit eye with Stage 2 PVR. (**A**) Fundus photo shows mild puckering of the medullary wings with distortion of blood vessels. There is no retinal detachment on H&E staining at 10X. (**B**) and 20X. (**C**) magnification. There is no staining of retinal surface membranes with vimentin (**D**,**G**), alpha smooth muscle actin (**E**,**H**) and glial fibrillary acid protein (**F**,**I**) at 10X or at 20X magnification.

H&E staining confirmed traction on the inner retina with folding of the outerretina in detached retina (Fig. 2B,C). Vimentin, a protein expressed by de-differentiated RPE cells, mesenchymal cells, Müller cells and other glial cells, was observed both in the subretinal space (Fig. 2D). Alpha smooth muscle actin (α-SMA), a marker for myofibroblasts derived predominantly from dedifferentiated RPE cells, was observed in both epiretinal membranes and in the subretinal space (Fig. 2E,H). Epiretinal membranes also stained positively for Glial fibrillary acid protein (GFAP), a marker of glial cells (Fig. 2F). Figure 3 shows the corresponding images for an eye with stage 2 PVR (mild pucker).

## Discussion

PVR is a complex disease with multiple stages in its evolution, involving many pathogenic pathways. To develop an effective therapeutic strategy, it is important to understand which pathways are involved and how to target them at the appropriate timing. Many models of PVR have been described over the years^9^. Most of the earlier models involved injection of fibroblasts into the vitreous cavity in an attempt to recreate the fibrosis seen in PVR. We now know that external fibroblasts are not involved in the PVR process^10^. To more accurately model the emergence of human disease, we chose an experimental model that mimicked the development of PVR after surgery, by inducing retinal detachment, release of RPE cells into the vitreous cavity, simulating a pro-inflammatory environment with cryotherapy and the injection of platelet rich plasma, avoiding the injection of any non-native cells. With this model, we found that PVR began as early as 2 weeks after surgery, with about 50% of eyes developing severe PVR with retinal detachment at week 4. Our PVR model was similar to that described by Goldaracena *et al*., in which they performed vitrectomy, retinotomy, cryotherapy and PRP injection. In contrast with our results, they found 100% rate of severe PVR with retinal detachment at week 4, with clinically apparent signs of PVR appearing at week 2 to 3^5^. Several variables in surgical factors could have contributed to this observed difference, including the extent of cryotherapy, size of retinotomies, amount of vitreous removed and number of RPE cells liberated into the vitreous cavity. To further optimize our surgical model, considerations are being made for controlling such factors.

In brief, the postulated pathogenic processes that occur during retinal detachment that lead to PVR are as follows^10^: 1. breakdown of the blood retinal barrier allowing microglia and macrophages to migrate into the subretinal space and the vitreous cavity where they release inflammatory cytokines, 2. release of RPE cells into the vitreous cavity, where they are stimulated by growth factors produced by a variety of cells including Müller cells to survive and proliferate, and 3. RPE cells then undergo mesenchymal transformation (epithelial mesenchymal transition, EMT) into fibroblast-like cells that then form contractile membranes on the surface of the retina, within the retina, and in the subretinal space. These PVR membranes may subsequently redetach the retina. It is clear from this sequence of events that a pro-inflammatory environment is a crucial first step for the initiation of the PVR disease process. Several inflammatory cytokines have been associated with PVR, including IL-6, IL-8, IL-10, IL-1β and interferon γ^11–16^. Our findings demonstrate that inflammation (as represented by elevation of IL-8) spikes within the first 2 weeks and continues to persist up to 4 weeks after induction of the PVR process. CRP and IFN-γ were significantly associated with PVR severity, suggesting that inflammation not only incites the PVR process, but also perpetuates its severity. Interestingly, the association of IFN-γ with PVR severity was independent of the duration post PVR induction. IFN-γ has been shown to cause RPE dysfunction by increasing the expression of the long noncoding RNA (lncRNA) BANCR. BANCR expression has been shown to elicit EMT like changes in cancer cells as well as ARPE-19 cells^17^.

Survival and proliferation of RPE cells are the next important step in the pathogenesis of PVR. Tumor protein 53 (TP53) suppression by activation of PDGF receptor α (PDGFR α) is a key event, allowing these cells to resist apoptosis and enhance proliferation. PDGFRα can be directly activated by PDGFs or indirectly by non-PDGFs. PDGFs have consistently been found to be elevated in both animal models of PVR as well as in human PVR^18,19^. However, it is the non-PDGF activation of PDGFRα that appears to be the major pathway of TP53 suppression as it circumvents the receptor downregulation mediated by PDGFs, allowing perpetual activation of PDGFRα^18^. In particular, VEGF promotes the non-PDGF pathway of activating PDGFRα by antagonizing PDGF-mediated dimerization of PDGFRs^20,21^, and anti-VEGF agents have been shown to completely suppress PVR development in animal models^21,22^. We found a significant trend for elevation of VEGF levels over 4 weeks in this study, and VEGF was also significantly associated with PVR severity independent of time from PVR induction. PlGF is a member of the VEGF family, bearing remarkable similarity in its three dimensional structure with VEGF isoform A^23^. We observed elevated levels of PlGF at 4 weeks post PVR induction, but how PlGF is involved in PVR pathogenesis is currently unknown.

Ang2, another member of the VEGF family, is a multifaceted cytokine involved in the regulation of angiogenesis and inflammation^24^. Increased levels of Ang2 has been found in vitreous samples from eyes with retinal detachment and have been suggested to contribute to PVR development^25^, but there are no studies to date describing an association with PVR. We found significantly elevated levels of Ang2 at week 4 post PVR induction. Interestingly, Ang2 has been implicated in lung cancer metastasis by increasing EMT^26^. Future research should investigate the possible role of Ang2 in promoting PVR via the EMT pathway.

There are limitations to our study. The sample size is small and may not be adequately powered to study small differences in cytokine and growth factor levels. This limitation is somewhat mitigated by vitreous sampling over many time points. There are many other cytokines that have been reported to be associated with PVR that we did not analyse in this study. However, we have chosen the most consistently reported cytokines and those that have been shown to play an important role in human PVR pathogenesis. Lastly, the study period of 1 month may not have been sufficient for PVR development in some of the study eyes. Nevertheless, having study eyes of different PVR severity did allow us to characterise the cytokine composition at the various stages of PVR development in the rabbit eye, which is the main objective of this study.

In conclusion, our study in a rabbit PVR model demonstrates intense inflammation beginning rapidly in the first 2 weeks after PVR induction which continues to be elevated up till 4 weeks post PVR induction. Shortly after the inflammatory phase, the growth factors IFN-γ, VEGF, PDGF-BB, PlGF and Ang2 likely support the survival, proliferation and EMT of RPE cells. The findings regarding Ang2 warrant further studies to better understand the role of this cytokine in PVR development. The findings presented here will guide future studies aiming to study inflammatory cytokines and growth factors as potential therapeutic targets for the treatment of PVR.

## Data Availability

The datasets generated during and/or analysed during the current study are available from the corresponding author on reasonable request.
